# Macros to Quantify Exosome Release and Autophagy at the Neuromuscular Junction of *Drosophila Melanogaster*

**DOI:** 10.3389/fcell.2021.773861

**Published:** 2021-11-15

**Authors:** Irene Sanchez-Mirasierra, Sergio Hernandez-Diaz, Saurav Ghimire, Carla Montecinos-Oliva, Sandra-Fausia Soukup

**Affiliations:** CNRS, Institut des Maladies Neurodégénératives, UMR 5293, Université de Bordeaux, Bordeaux, France

**Keywords:** autophagy, autophagosome, *Drosophila*, exosome release, quantification, synapse, neuromuscular junction

## Abstract

Automatic quantification of image parameters is a powerful and necessary tool to explore and analyze crucial cell biological processes. This article describes two ImageJ/Fiji automated macros to approach the analysis of synaptic autophagy and exosome release from 2D confocal images. Emerging studies point out that exosome biogenesis and autophagy share molecular and organelle components. Indeed, the crosstalk between these two processes may be relevant for brain physiology, neuronal development, and the onset/progression of neurodegenerative disorders. In this context, we describe here the macros “Autophagoquant” and “Exoquant” to assess the quantification of autophagosomes and exosomes at the neuronal presynapse of the Neuromuscular Junction (NMJ) in *Drosophila melanogaster* using confocal microscopy images. The *Drosophila* NMJ is a valuable model for the study of synapse biology, autophagy, and exosome release. By use of Autophagoquant and Exoquant, researchers can have an unbiased, standardized, and rapid tool to analyze autophagy and exosomal release in *Drosophila* NMJ.

Code available at: https://github.com/IreneSaMi/Exoquant-Autophagoquant

## Introduction

The brain is one of the most complex and dynamic organs in the body. The most common brain cells are neurons and glia. Each of them expresses different proteins according to their nature, function, or needs. Different stimuli such as neuronal activity, glucose, calcium, etc., can also affect protein expression, localization, and even function. This modulation can result in memory consolidation and synaptic plasticity (Rosenberg et al., 2014), synaptogenesis (Kriegstein and Schmitz, 2003), or differentiation (Nazir et al., 2018). The pattern of expression can also be modulated by genetic or environmental factors indicating a change in the function or making evidence of a pathological state in the neuron that ultimately can affect brain function. Protein and lipid dysfunction and accumulation of aggregate-prone or dysfunctional proteins (due to proteostasis dysfunction) are commonly affected and may be at the root of many neurodegenerative diseases [reviewed in (Hernandez-Diaz and Soukup, 2020)] such as multiple sclerosis (Mandolesi et al., 2015), Alzheimer’s (Chen et al., 2019) or Parkinson’s disease (Imbriani et al., 2018).

Synapses are highly specialized neuronal structures responsible for transmitting information from one neuron to one or more targets cells (neuronal and non-neuronal cells). The biology of the synapse is an emerging but challenging field due to their location, abundance, and relatively small size (especially in mammals (∼1–2 µm) (Glasgow et al., 2019). Synapses can transmit information using electrical or chemical signals. Chemical synapses relay on the secretion of neurotransmitters by a process known as synaptic vesicle release. Besides synaptic vesicles, the release of intracellular vesicles containing active biomolecules such as exosomes or ectosomes by cells is gaining attention, especially in the context of brain (dys)function. Neuromuscular Junction (NMJ) is a valuable model to study the pre-synapse as the neuron is clearly differentiable from the muscle (Rodríguez Cruz et al., 2020). In 1791, Luigi Galvani described the electrical properties of the neuromuscular connection (Galvani, 1791) and Bernard Katz described the quantal nature of neurotransmitter release (Fatt and Katz, 1952) using the neuromuscular junctions of the frog. A more recent example is the *in vivo* study of synaptic transmission using the NMJ in Zebrafish (Brehm and Wen, 2019). In *Drosophila*, larval NMJ has been widely studied to understand not only general synaptic development (Ruiz-Cañada and Budnik, 2006; Chou et al., 2020) and morphology (Nijhof et al., 2016) but also to investigate the regulation of glutamatergic synapses (Petersen et al., 1997). A unique feature of the *Drosophila* NMJ synapses is their big size (between 2 and 5 µm in diameter) (Knodel et al., 2014), which allowed initial research without the need for super-resolution imaging techniques. The synapse at the NMJ of *Drosophila* is a series of rounded expansions of the membrane, also called boutons that shape the motor-neuron presynapse surrounded by the muscle subsynaptic reticulum.

Previous studies showed that synaptic autophagy, a catabolic mechanism, is induced and locally controlled at the presynaptic compartment of the NMJ (Soukup et al., 2016; Okerlundk et al., 2017; Vanhauwaert et al., 2017). The regulation of autophagy is critical for synaptic homeostasis, development and neuronal survival (Shen and Ganetzky, 2009; Soukup et al., 2016; Vanhauwaert et al., 2017; Nikoletopoulou and Tavernarakis, 2018; Lieberman and Sulzer, 2020). Research on autophagy relies on the quantification of autophagy markers (LC3/Atg8-I; LC3/Atg8-II) in western blot or on quantification of autophagosomes in confocal microscopy [see autophagy guide (Klionsky et al., 2021)]. Publications often quantify manually the number of mature autophagosomes, defined as LC3/Atg8 positive clusters, or even the number of immature pre-autophagosomes, as ATG18a/WIPI2 positive clusters (Soukup et al., 2016; Okerlundk et al., 2017; Vanhauwaert et al., 2017; Guo et al., 2019).

**FIGURE 1:**
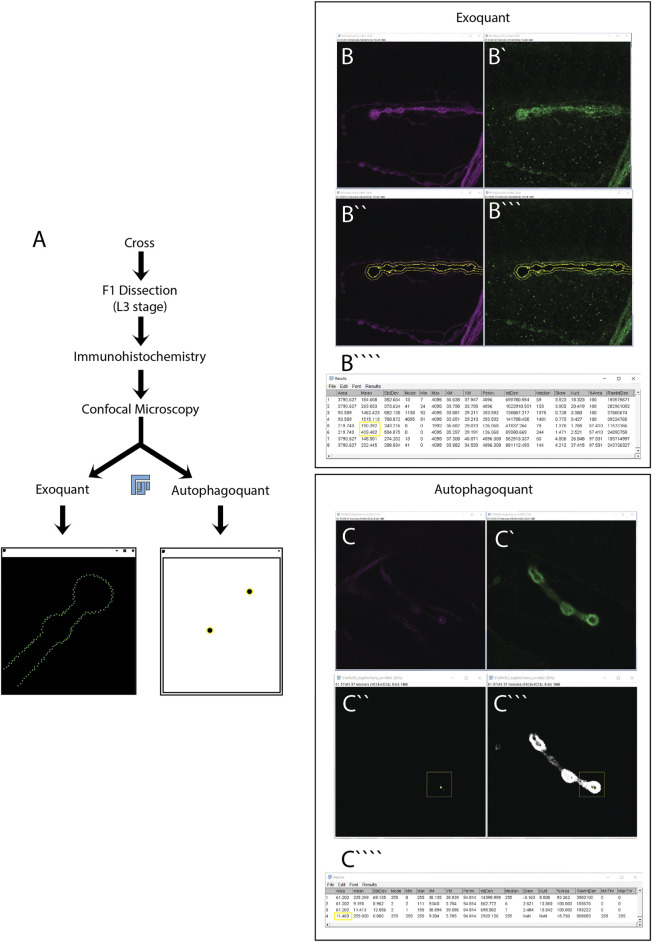
Experimental workflow for the quantification of exosome release and autophagy using the Exoquant and Autophagoquant macros. **(A)** Parental *Drosophila* adults (F0) with the correct genotype are cross using a 3:1 female to male ratio at 21°C. F1 *Drosophila* larvae with the desired genotype are dissected in L3 stage. A critical process in the dissection step is to maintain the nerves intact. Dissected larvae are fixed with PFA before immunohistochemistry using the appropriate antibodies. After the immunohistochemistry, larvae are mounted and imaged using confocal microscopy. Two-channel confocal representative images of *Drosophila* NMJ are selected for quantification of exosome release **(B,B’)** or autophagy **(C,C’)**. Using the macros, you will obtain these windows for Exoquant **(B”,B”’)** or Autophagoquant **(C”,C”’)** macros. A final table with the main parameters to quantify exosome release **(B””)** or autophagy **(C””)** is obtained. The parameters used to do the quantification in this paper are highlighted in a yellow box.

Not only autophagy but also exosome release at the NMJ has been shown to be crucial for synapse development, synaptic plasticity and synaptic homeostasis (Korkut et al., 2009; Korkut et al., 2013). Exosome-mediated synapse development relies on Wingless (Wg) signaling to the postsynaptic site. A function that is conserved in mammals by the Wg homolog, Wingless-type (Wnt) (Ahmad-Annuar et al., 2006; Gross et al., 2012; Hooper et al., 2012; Koles et al., 2012; Ekström et al., 2014). The transmembrane protein Evenness interrupted (Evi/Wls) transports Wg on exosomes to the postsynaptic site where Wg binds to its receptor Frizzled2. Several publications report also quantifications of exosome release at the *Drosophila* NMJ (Korkut et al., 2009; Koles et al., 2012; Korkut et al., 2013; Lauwers et al., 2018), but the manual methods used here are not extensively described. Manual quantification is time-consuming and involves several individual decisions (i.e., set up of the threshold, area of interest, etc.) which make replication challenging. In this paper, we described and report two different macros built with FIJI (an image processing package of ImageJ: an open-source image processing program) that serves as a powerful toolbox to quantify autophagosomes (protein clusters) and exosomes (extrasynaptic protein intensity) at the *Drosophila* NMJ. An overview of the main working steps of quantification and the macros can be found in (Figure 1A). The first automatic method quantitatively assesses the amount of fluorescence intensity within a personalized distance range from the terminal axon of motor neurons in *Drosophila* using fluorescence microscope images (Figures 1B–B””). The second automatic method quantifies the number, size, and fluorescence intensity of protein clusters/Atg8 positive dots (representing autophagosomes) inside the NMJ (Figures 1C–C””). This method allows the user to discriminate protein clusters by size, using also fluorescence microscope images.

We validated our automated quantification method to quantify autophagosomes by applying this method in an amino-acid starvation assay, a standard method to induce autophagy. Likewise, we use Hsp83 mutation, which has already been shown to impact exosome release, to validate our automatic quantification method to analyze exosome release. This study is the first that completely automatized the task without the researchers decision-making intervention, making it highly reproducible over time and providing new opportunities to compare the data in an objective/unbiased and reproducible way.

## Material and Methods

### Software

1) ImageJ/FIJI (https://imagej.net/Fiji) (Schindelin et al., 2012)
2) Exoquant surrounding detector macro for ImageJ (attached as Supplemental Material in this paper and also available in the GitHub: www.github.com/IreneSaMi)
3) Autophagoquant dot detector macro for ImageJ (attached as Supplemental Material in this paper and also available in the GitHub: www.github.com/IreneSaMi)
4)Excel (Microsoft https://www.microsoft.com/es-ww/microsoft-365/excel), Prism 9 (Graphpad https://www.graphpad.com/scientific-software/prism/) and Illustrator 2021 (Adobe, https://www.adobe.com/es/products/illustrator.) Alternatively, you can use R (The R Foundation, https://www.r-project.org/)

### Software and Image Availability in Public Repositories

We uploaded the macros to the public repository GitHub (www.github.com/IreneSaMi) and the images we used for the macros under www.zenodo.org (user name: IreneSaMi).

### Equipment

Confocal microscopy (TCS SP5, Leica Microsystems) with a Leica HCX PL APO lambda blue 63×/1.40–0.60 OIL UV objective.

### Fly Housing

Flies used in this article were grown on standard cornmeal and molasses medium supplemented with yeast at 21°C.

**Table:** 

*Drosophila melanogaster* lines	Source	Identifier
w [1118]	Bloomington *Drosophila* Stock Center	BDSC:3605; FlyBase: FBal0018186
w [*]; Hsp83 [e6A]/TM6B, Tb [1]	Kyoto Stock Center	Kyoto DGGR: 108,372; FlyBase: FBst0307017
w; UAS-Evi-GFP	M. Boutros Bartscherer et al. (2006)	N/A
w; P{GawB}D42	Bloomington *Drosophila* Stock Center	BDSC:8816; FlyBase: FBti0002759
w; UAS-Atg8mCherry,OK6Gal4	T. Neufeld Scott et al. (2004)	N/A
w; P{GawB}OK6	Bloomington *Drosophila* Stock Center	BDSC:64,199; FlyBase: FBti0023258

**Table:** 

Antibodies	Source	Identifier	Dilution
Chicken anti-GFP antibody	AVES Lab	GFP-1020	1:1000
Rabbit polyclonal anti-HRP	Jackson ImmunoResearch	Cat#323-005-021, RRID:AB_2314648	1:500
Mouse monoclonal anti-DLG antibody	Developmental Studies Hybridoma (DSHB) Bank	Cat#4F3, RRID:AB_528,203	1:500
Rabbit anti-mCherry	NOVUS	NBP2-25157	1:500
Alexa Fluor 488 Goat anti-chicken antibody	Invitrogen	Cat#A-11039, RRID:AB_142,924	1:1000
Alexa Fluor 488 Goat anti-mouse antibody	Invitrogen	A28175	1:1000
Alexa Fluor 555 Goat anti-rabbit antibody	Invitrogen	Cat#A-21429, RRID:AB_141,761	1:1000

### Larva Preparation and Immunohistochemistry

Male and female third instar larvae were used and distributed randomly among the experiments as previously described (Soukup et al., 2016; Lauwers et al., 2018). Larvae were dissected “in fillets” on syrgard plates in HL3 buffer (110 mM NaCl, 5 mM KCl, 10 mM NaHCO3, 5 mM HEPES, 30 mM sucrose, 5 mM trehalose, and 10 mM MgCl2, pH 7.2). For immunohistochemistry, larvae were fixed for 20 min in a 4% PFA/HL3 solution. Larvae fillets were permeabilized with 0.4% Triton/PBS (PBT) for 1 h and pre-blocked with 10% NGS-PBT for 30min at RT. Samples were incubated with the primary and secondary antibodies in 10% NGS-PBT overnight at 4°C and for 1.5 h at RT, respectively. To analyze exosome release, we used the anti-GFP (to label the EVI-GFP/Exosome marker) and anti-HRP (that recognize neuronal membrane/presynaptic marker). For the autophagy experiments, samples were stained with anti-mCherry (to label the Atg8-mCherry/Autophagosome marker) and anti-DLG (to label the surroundings of the synapse). Samples were mounted on Vectashield (Vector Laboratories) and kept at 4°C until imaging.

### Starvation Assay

Starvation assays were performed as previously described (Soukup et al., 2016). To induce autophagy we subjected the flies to amino acid deprivation (also called starvation). For this assay, larvae with low population density were grown on standard food supplemented with yeast paste at RT (21°C) For amino-acid starvation, early third instar larvae were placed on Petri dishes containing 20% sucrose and 1% agarose during 3–4 h at 25°C, followed by rapid dissection of the larvae.

## Procedure

### Image Acquisition

The macros Exoquant and Autophagoquant work on confocal microscope images. However, these macros could potentially quantify other types of images with similar characteristics just by making some adjustments. The images should have been optimized to have a high signal-to-noise ratio. The ideal confocal images should not contain pixel saturation as this will not allow quantification. However, saturation is not a problem in areas that are not the object of the quantification. If you want to compare between different images, it is highly recommended -if not mandatory- to maintain identical acquisition settings for all the images and conditions.

In particular, the settings used to obtain the data were: image size 1024x1024 pixels 12 bits. The pinhole size was fixed at 1 AU. The laser settings might vary according to the microscope, antibodies and samples. Five larvae per genotype/condition and 4 NMJs per larvae, were taken. To guarantee representability, each NMJ image was taken from a different segment (A2, A3, or A4) and two from each side (left-right) of the larvae.

### Macro Installation

#### Procedure 1

Copy the Autophagoquant.ijm or Exoquant.ijm in the Fiji.app/macros folder.

Select Plugins > Macro > Install … and search the file Autophagoquant. ijm or Exoquant. ijm and click open.

Select Plugins > Macros and the name of the file (Autophagoquant or Exoquant will appear on the Macros display menu). You will need an open image to run the macros.

#### Procedure 2

Select the.ijm file and drag it into the Fiji window. This will open the editor of macro window showing the script and this option allows editing the parameters. Then, open your image and press Run.

### User Adjustments

The output parameters in the result table can be personalized in lines 4 (Exoquant) and lines 16, 36 (Autophagoquant). The color of the foreground (white), background (black), and selection (yellow) are set up and should not be modified because the “clear” option uses the color of the background, and modification of this parameter will therefore affect the results.

You can order the channels in lines 10, 13, 17, 28, 31, 37, 40, 46, 49 (Exoquant) and in lines 9, 19, 20, 26, 44 (Autophagoquant). Channel 1 is set up to be the channel for quantification and channel 2 is for the mask -membrane marker-(Exoquant and Autophagoquant).

### Function of the Macros

#### Exoquant

**FIGURE 2:**
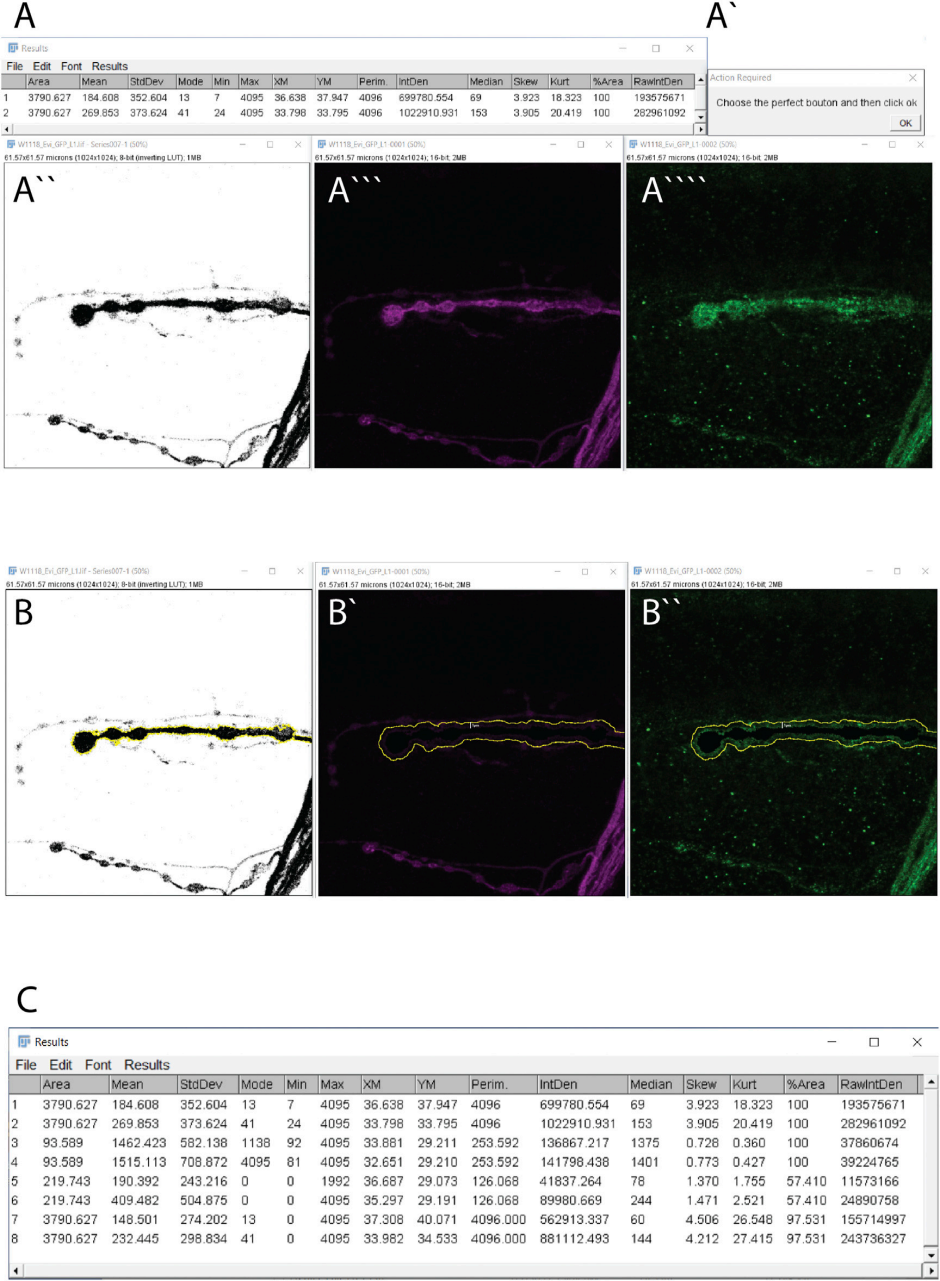
Exoquant macro working steps and results. **(A–A””)** The four windows results are obtained automatically when you run the macro. **(A)** The two lines with measurements from the raw data of the two channels. **(A’)** The action window to detect the shape of the synapse. **(A’’)** Image after the application of the Otsu algorithm to automatically detect the presynaptic membrane marker (HRP channel/outline). **(A‴)** Original image of HRP and **(A”’’)** Evi-GFP signal. **(B,B”)** These windows appear after confirming the synapse shape. **(B)** The neuronal membrane outline (yellow) that was selected in the previous step. **(B’)** The neuronal membrane channel with the selected synapse in black with a value of 0 and in yellow is the enlargement of 1 μm from the presynapse (imposed for clarity but will not appear). **(B”)** The exosome signal with the neuronal presynapse selected in black with value 0 and in yellow the enlargement of 1 μm from the neuron (imposed for clarity but will not appear). **(C)** The result window.

1) Open a confocal image using Fiji (or ImageJ) software from which you want to quantify the amount of fluorescence surrounding the presynaptic NMJ bouton.
2) Adjust the extracellular distance from the outer membrane of the bouton you want to measure in lines 38 and 42. Evi-GFP positives extracellular vesicles ranging between 30 and 150 nm (hereby “exosomes”) are traveling inside the subsynaptic reticulum (SSR) of the postsynaptic side. Therefore, the default extracellular distance to quantify the exosomes is 1 μm (corresponding to the SSR diameter).
3) Launch Exoquant Macro from Plugins > Macro > Exoquant.
4) The macro opens four windows [Figure 2A, A’, A’’, (A’’’-A’’’’ channel 1 and 2 in the same window)] corresponding to (Figures 2A’’’–A’’’’) your confocal image, (Figure 2A), the raw image measurements in both channels (row 1 for the membrane marker and row 2 for the protein of interest) (Figure 2A”) One image is a duplicate from the membrane marker channel in which the Otsu threshold method is applied to highlight the shape of the bouton (the mask) in black. (Figure 2A’). A pop-up window that requires an action indicating “Choose the perfect bouton” will appear. This action initiates the auto-Threshold (v1.17.2) using the Otsu method (Otsu, 1979). We empirically tested 16 auto-threshold methods in FIJI such as Default, Huang, Huang2, Intermodes, IsoData, Li, MaxEntropy, Mean, MinError(I), Minimum, Moments, Percentile, RenyiEntropy, Shanbhag, Triangle and Yen (https://imagej.net/Auto_Threshold) and the Otsu method obtained the best results in our datasets. The Otsu method optimizes the threshold for each image.
5) To “Choose the perfect bouton” for the action window, click on the bouton within the threshold window to define the region of interest (ROI, in yellow) using the wand tool (see specification in macro code (line 23) (Figure 2A”) and then click “ok” in the action required window to finish the task.
6) You will obtain 1) the thresholded image with the ROI highlighted in yellow (Figure 2B), 2) the window of your confocal image containing the shape of the bouton in black with a value of zero (Figures 2B’,B”, in yellow is highlighted 1 μm from the bouton**)** and 3) the results window (Figure 2C).
7) The results window contains 8 rows. Rows 1 and 2 have been previously described. Rows 3 and 4 correspond to measurements from inside the perimeter of the selected area/bouton 3) in the mask channel and 4) in protein-of-interest-channel. Rows 5 and 6 show data from the enlarged extracellular area after the area of the inside of the cell (data from raw 3) is set to zero in (5) the mask-channel and row 4 in (6) the protein of interest-channel. Thus, intensity values in row 6 represent the amount of Evi-GFP positive exosomes in the extracellular part of that particular synaptic bouton. Rows 7 and 8 contain the measurements of the full image after subtraction of the data from row 3 in (7) the mask-channel and row 4 in (8) the protein-of-interest-channel.

#### Autophagoquant

**FIGURE 3:**
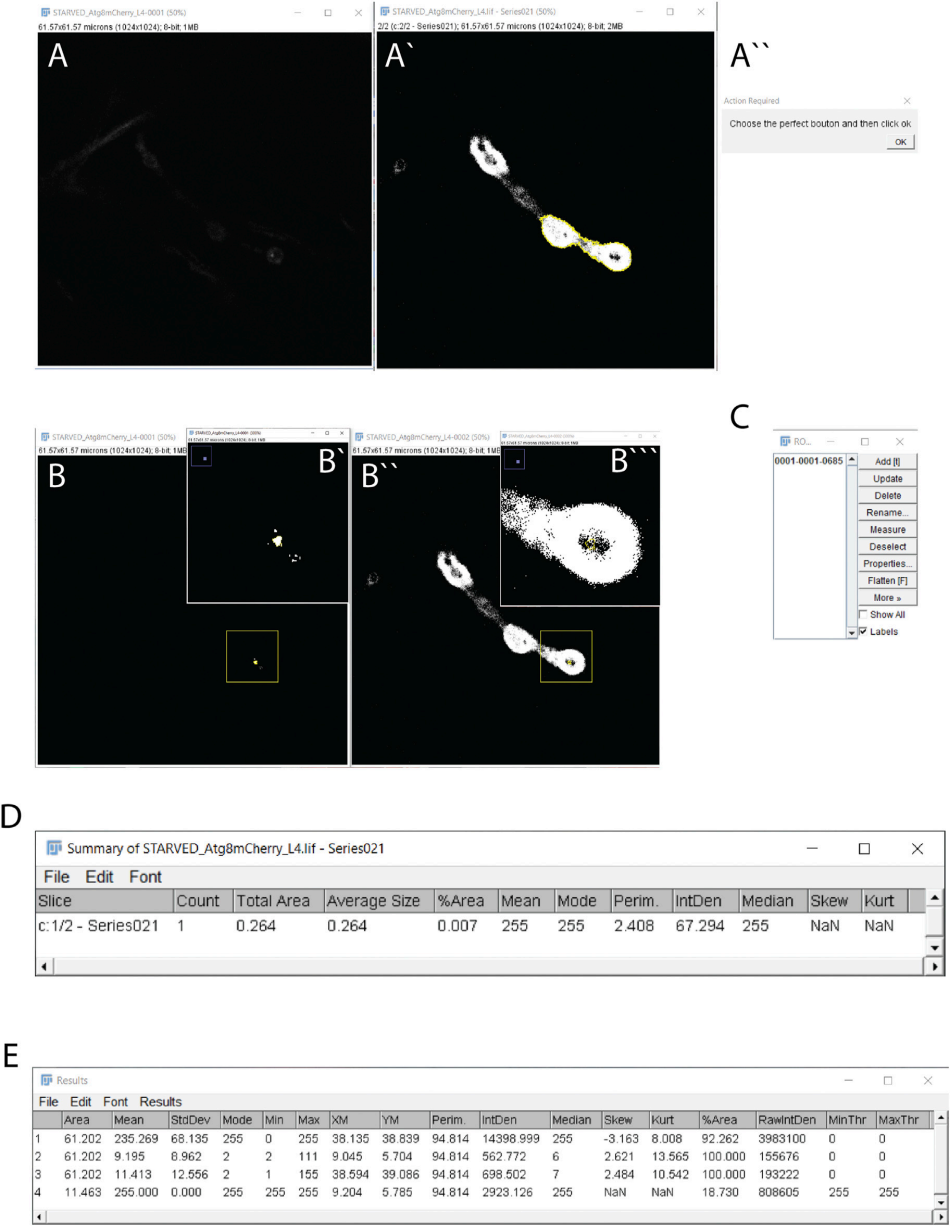
Autophagoquant macro working steps and results. **(A–A”)** The windows are obtained automatically when you start running the macro. **(A)** The original Atg8-mCherry confocal image (protein-channel) in 8-bit and **(A`)** the DLG channel is marking the postsynaptic compartment (DLG) after thresholding by the Otsu algorithm. The yellow outline appears when you select the ROI by using the wand tool in this window. **(A``)** The window to confirm (clicking OK) to select the shape of your neuron. **(B,B’’)** After the “choose your perfect bouton” action, these windows appear. **(B,B’)** The Atg8-mCherry channel (protein-channel) is thresholded inside the DLG selection. Note, in white appears only the pixels with a value higher than the mean plus 4 times the standard deviation. If the size of the pixel connected is inside the range you set (0.09–0.5 μm) it will appear with a yellow outline. **(B’’,B’’’)** The DLG channel is thresholded and its magnification with the dot to see them clearly inside the DLG channel. **(C)** Window with the ROI manager in which all the dots selection (autophagosomes) will be stored **(D)** The summary window in which all the dots will be counted and characteristics of all of them together will be taken into account **(E)** The result window.

1) Open a confocal image using Fiji (or ImageJ) software in which you want to quantify the protein clusters (like Atg8 positive dots/autophagosomes) inside the bouton. The confocal image is automatically transformed into an 8-bit image to allow protein cluster quantification.
2) The results table must be empty before running the macro. The macro will automatically clean the results table before each new image is quantified. The reason is that the macro will always use the data from raw 2 in the results table to determine the mean and the standard deviation of the current image.
3) Personalization of Parameters:
  a) Particle size. Particles are classified and quantified according to their size (min and max measured as μm or pixel size). Particles that are not in the user’s defined range are discarded from the final quantification. The range for autophagosome quantification has been empirically determined and is set up in the range of 0.09–0.5 μm in macro line 52. Previously, Soukup et al. performed correlative light and electron microscopy (CLEM) in *Drosophila* NMJ synapses and determined that Atg8 positive autophagosomes had a diameter ∼100 nm in electron microscopic images (Soukup et al., 2016). However, some treatments, mutations, and/or conditions could alter the size of autophagosomes (Jin and Klionsky, 2014; Kjos et al., 2017; Yamamoto et al., 2020). To adjust the size interval to these particular cases we recommend drawing a straight line through the center of the fluorescence cluster of the autophagosome marker with the straight tool. Then, use the plot profile tool (Analyze > Plot profile) and measure the base of the curve. Repeat this process several times with autophagosomes from different samples to obtain an empirically optimized size range within your experimental dataset.
  b) Users can also adjust circularity from 0 to 1 in line 52. Selecting 1 involves selecting perfect circular clusters according to the formula *4pi(area/perimeter* ^*2*^ *)*. Empirically, we have determined that the circularity of Atg8-mCherry positive fluorescence clusters are not consistent and therefore, we recommend setting circularity to ‘0’ to quantify autophagosomes.
  c) The difference between the fluorescence intensity of the cluster (autophagosome) and the rest of the synaptic compartment can be modified in macro line 47. Normally modifying the parameter standard deviation of the mean fluorescence intensity (set up at 4).
  d) The filter median with a radius of 1 pixel is chosen to reduce the noise, replacing the pixel with the median value of the surrounding pixels. This value was empirically tested against the mean filter and 1-5 pixel range of radius value. The users can modify this parameter in macro lines 21 and 40.
  e) The distance to discriminate close or adjacent clusters as single or multiple objects. To measure synaptic autophagy, this value is empirically set at 0.5 μm but users can modify this value in line 58.
4) Launch Autophagoquant Macro from Plugins > Macro > Autophagoquant.
5) You will have two windows (Figures 3A,A’ in one stack and 3A’’ in another**)** corresponding to 1) your confocal image in 8-bit (Figure 3A) with the DLG channel after applying a threshold using the Otsu algorithm (Figure 3A’). In this case, we label the muscle postsynaptic compartment using the DLG protein. 2) A window that requires an action indicating “Choose the perfect bouton and then click Ok” (Figure 3A”).
6) Select the area containing your ROI (Figure 3A’).
7) To detect the autophagosomes, the macro will automatically apply a threshold equal to the mean intensity plus four times the standard deviation of the ROI in which the filter is applied (the user can change this threshold setup in macro line 47). If these pixels are physically connected and form a dot with a size ranging from 0.09 to 0.50 μm, they are taken into account and will appear in the ROI window.
8) Finally, you will have four windows corresponding to 1) your confocal image after applying the automatic threshold for the autophagosome and the second channel according to the Otsu method (Figures 3B, B”), 2) the ROI manager window that will identify the autophagosomes within the ROIs. If there is more than 1 autophagosome, then the macro will test if they are different or unique autophagosomes based on the distance parameter set in 3 c). In our particular case, this value is set at 0.5 μm. If the fluorescence clusters lay within the limit, the macro will merge them into a single dot. This will be done with all the dots and with new dots resulting from the merge (Figure 3C) and this data can be saved for the posterior quantification of the fluorescence intensity. The summary table opens with all the dots within the size range counted in the confocal image:total area, average size, % of area, mean and mode (which should be 255), perim, intden, median, skew, and Kurtosis (Figure 3D). The results window shows the measurements from the selected bouton (Figure 3E).
9) The results window contains 4 rows. 1) The second channel (here we used the postsynaptic marker DLG) once it is thresholded with the Otsu method, 2) the area selected but in the channel 1 once is done the filter median with the radius 1, 3) the area selected in channel 1 without the filter and 4) after Otsu thresholding the area that is above the threshold of the protein channel.

## Data Analysis

### Exoquant

**FIGURE 4:**
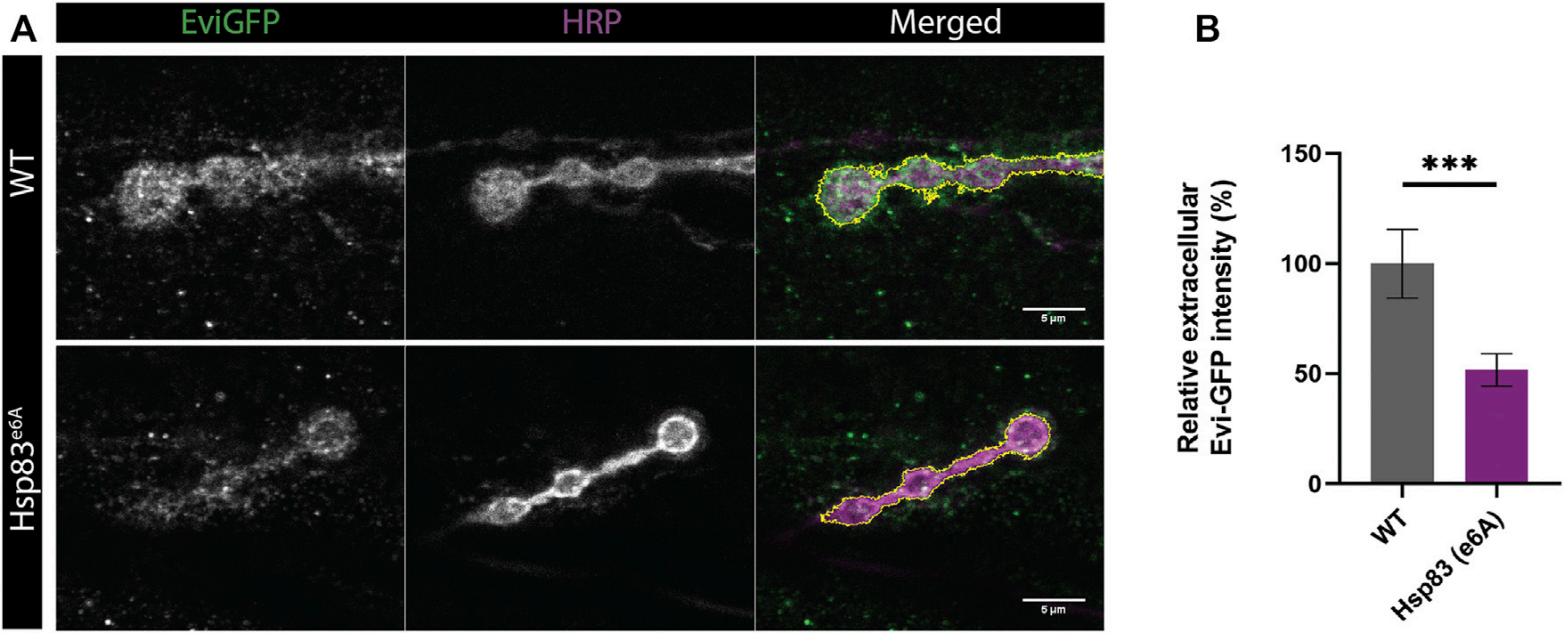
Results obtained using Exoquant macro. **(A)** Representative images of neuromuscular junction (NMJ) boutons from wild type (WT) and Hsp83^e6A^ mutants expressing Evi-GFP (green). The presynaptic compartment is labeled with anti-HRP antibody (HRP). Merge image highlights in yellow the presynaptic membrane. **(B)** Quantification of exosome release using Exoquant shows lower relative Evi-GFP fluorescence in Hsp83^e6A^ mutants compared to wild-type animals. Values were normalized to wildtype. n = 18 (wild-type) and n = 28 (Hsp83^e6A^) image fields per condition. Data were analyzed using a student t-test (two-tailed) for normal distributions. Normality was analyzed with D’Agostinothe -Pearson Omnibus test. ***p < 0.001, data shown as mean ± SEM.

Reducing the levels of the protein Hsp83 using the Hsp83^e6A^ hypomorphic mutation decreases exosome release and promotes multi vesicular bodies (MVBs) accumulation at the presynapse of *Drosophila* NMJ, while overexpression of Hsp83 in this mutant background rescues exosome release (Lauwers et al., 2018). We tested if our macro could automatically quantify exosome release and reproduce the published results. Thus, our macro detects a reduction of exosome release in the mutant Hsp83^e6A^ in the neuronal compartment of the NMJ (Figures 4A,B). However, in the publication they manually quantify the ratio between Evi-GFP intensity internal and external. This ratio may be affected by conditions that alter protein expression at the presynaptic compartment and therefore could complicate the comparison between conditions. Our macro directly quantifies the extracellular Evi-GFP signal in the SSR, where the Evi positive exosomes travel once released. In this method, we calculate the exosome release area (up to 1 μm) *via* division with the same area in the HRP signal, thus allowing direct comparison between different genotypes.

### Autophagoquant

**FIGURE 5:**
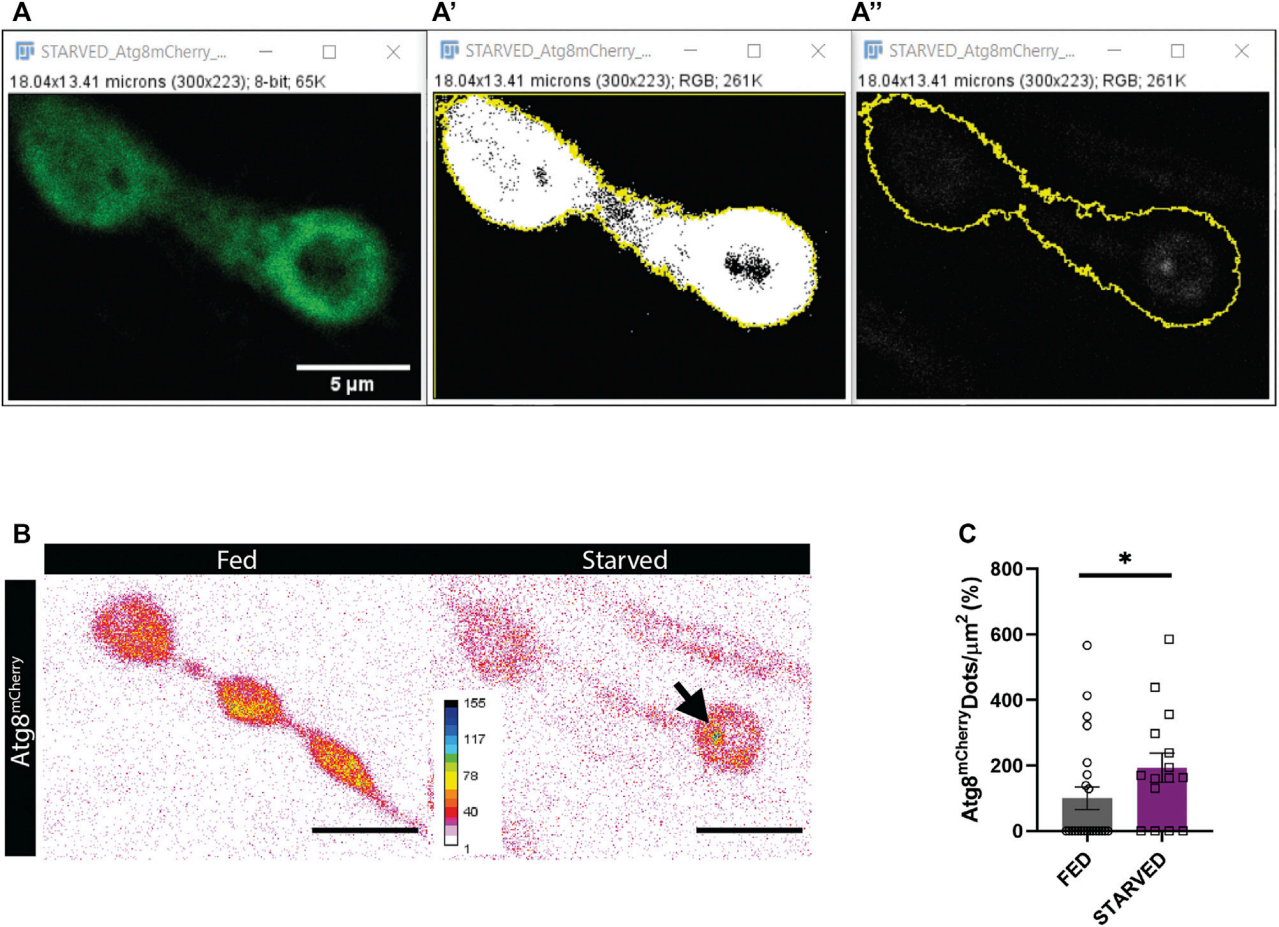
Results obtained using Autophagoquant macro. **(A,B)** NMJ boutons of *Drosophila* larvae expressing the autophagosome marker Atg8-mCherry (LC3 in mammals) under the control of the motorneuron promotor D42 (D42 -Gal4 > Atg8^mCherry^). **(A)** Confocal image of DLG staining and **(A’)** threshold DLG signal to determine synapse outline. **(A’’)** Confocal image of Atg8-mCherry signal with synapse outline in yellow obtained from thresholding the DLG signal. **(B)** Representative images of Atg8-mCherry signal are displayed in inverted 16 colors LUT for fed and starved condition. **(C)** Quantification with Autophagoquant shows that more autophagosomes are formed after starvation. Values were normalized to wildtype in the fed condition. *n* = 22 (fed) and *n* = 16 (starved) image fields per condition. Data were analyzed Mann-Whitney (two-tailed) for not normally distributed data. Normality was analyzed with D’agostino-Pearson Omnibus test *p < 0.05, data shown as mean ± SEM.

Amino-acid starvation is the most widely used paradigm to induce autophagy. Upon 3-4 h of amino acid starvation in third instar *Drosophila* larvae, the number of autophagosomes increases in the presynaptic compartment of the NMJ (Soukup et al., 2016). We completely automatized the task and reached the same conclusion (Figures 5A–C). Among the advantages of using the macro, you can obtain the intensity and the size values among other parameters if the objective is to compare between conditions. Automatic quantification requires high-quality confocal images. We recommend selecting images where NMJs are not disturbed by other structures such as nerves, trachea, fat body, or salivary glands if these structures are kept during the dissection phase.

For statistics, a minimum of 15 images from different boutons and at least 3 different animals are recommended.

## Discussion

**FIGURE 6:**
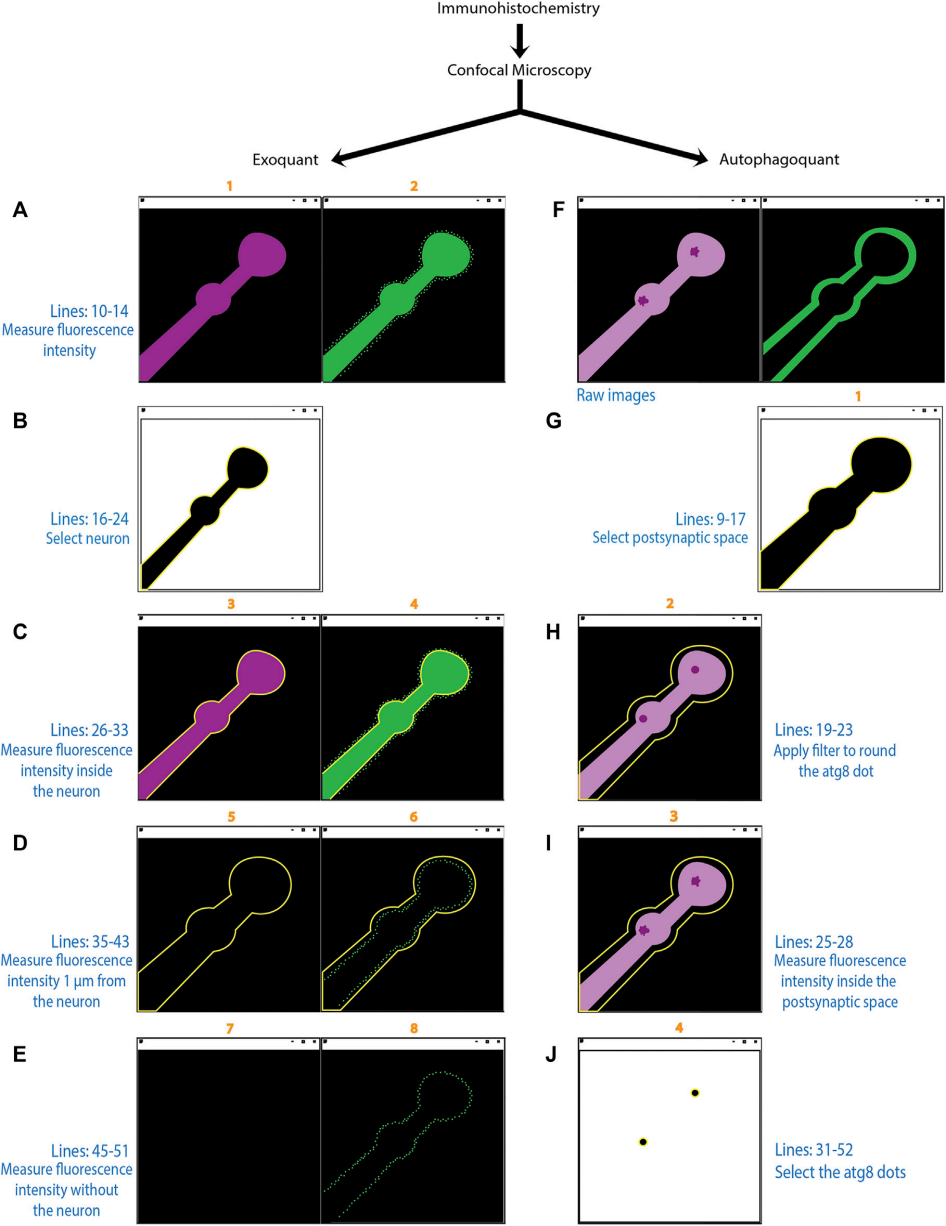
Diagram showing the workflow of Exoquant and Autophagoquant macros. Schematic representation showing the function of the two macros in the experimental design. The numbers over the individual pictures (in orange) corresponds to the line in the result window. The precise lines of the code (in blue) and a brief description of the underlying algorithm is described for each step of the macros (Exoquant: A-E; Autophagoquant: F-J). The “Exoquant” workflow shows the two channels from the original image obtained in the confocal microscope, in magenta the membrane marker (HRP) and in green the protein channel **(A)**. Fluorescence intensity is measured and stored in lines 1 and 2 of the results table. The membrane channel was thresholded with the Otsu method and highlighted in yellow after the selection with the wand tool **(B)**. The next step shows the shape of the membrane in both raw images **(C)**. Fluorescence is measured inside on both channels and correspond to lines 3 and 4 in the result table. The enlargement of 1 μm from the membrane and the measurements inside this area correspond to lines 5 and 6 in the result window **(D)**. Lines 7 and 8 in the result window correspond to the raw data before the selection has been made **(E)**. In the “Autophagoquant” workflow, the raw data are coming from the confocal microscope with two channels, in magenta is the protein that concentrates in dots and in the second channel is the surrounding marker DLG **(F)**. The surrounding channel is thresholded with the Otsu method and highlighted in yellow after the selection is made **(G)**. The dot protein channel in which you made the median filter and quantified the intensity inside the shape of the surrounding channel and correspond to line 2 in the result table **(H)**. Measurements of the inside of the shape marker from the raw data corresponding to line 3 in the result table **(I)**. Image in **(J)** is the result of the threshold filtered image with the number resulting from the mean intensity plus 4 standard deviations. If the resulting dots are in the range of 0.09 to 0.5 μm they will be added to the ROI manager and counted as a dot. If that is not the case, they will not be highlighted in yellow.

In this methodological article, we describe two macros, Exoquant and Autophagoquant, which improves the standardization in quantifying exosome release and autophagy processes at the synapse. We summarised the workflow for both macros indicating with numbers the steps from which we extract the measurements corresponding to the lines in the results tables (Figure 6). These macros implement the Otsu algorithm to automatically detect protein clusters presynaptic compartments or proteins levels surrounding this compartment in a reproducible and researcher-independent way. These macros also solve the difficult task of determining the correct threshold parameter question. However, these macros have limitations in quantifying images with a low signal-to-noise ratio or when additional larvae structures, such as the trachea, fat bodies, or nerves, are located close to the ROI. Regarding cluster quantification, high-quality images are preferred, as images showing immunostaining artifacts could interfere with cluster identification. We advise manually optimising parameters like cluster size or intensity according to the user’s experimental settings and design. Images could also be automatically opened for quantification, but we highly recommend observing the image before starting quantifications to discard low-quality images. We used the *Drosophila* NMJ to develop an automatized and objective FIJI-macro to quantify fluorescence intensity inside, outside, and in aggregates, in a reproducible way. We test the method against validated and published data and the two macros were able to detect the expected differences in exosome release and the number of autophagosomes with the benefit of increased reproducibility by reducing the researcher’s bias. In conclusion, this method allows a consistent method of comparison between experiments and researchers to quantify exosome release and autophagosomal formation at the *Drosophila* larval NMJ.

The user can modify Exoquant macro parameters to perform different quantification strategies. For instance, quantification of exosome release as the ratio between Evi-GFP fluorescence intensity outside and inside the presynaptic compartment is possible (Lauwers et al., 2018), but the user should avoid pixel saturation. Exoquant also provides information about the asymmetry of the distribution (skewness) that indicates if your protein is equally distributed and if the intensity follows a normal distribution. This macro could also measure the background after different immunofluorescence protocols comparing the total fluorescence intensity of your image before and after subtracting the intensity within your ROI (result window lines 7 and 8).

For the autophagosomes or protein clusters, the macro Autophagoquant calculates the centroid (the point at the centre of the selection) and you could, for instance, calculate the distance to the plasma membrane. This macro provides the user with values such as the intensity and size of the cluster if you keep the ROI window (Figure 3C) and open again the image. The macro can also calculate the area occupied by the clusters in relation to the area of the neuron and if the intensity is equally distributed within this area. However, these secondary uses of Autophagoquant are neither validated nor covered by this method article and should be implemented and validated by the users.

Furthermore, Autophagoquant could be used to quantify other organelles like synaptic mitochondria and endosomes by adjusting the detected particle size.

## Data Availability

The original contributions presented in the study are publicly available. This data can be found here: https://github.com/IreneSaMi/Exoquant-Autophagoquant (DOI 10.5281/zenodo.5576217).
