# Supplementary material for: Macros to Quantify Exosome Release and Autophagy at the Neuromuscular Junction of Drosophila Melanogaster
Source: Front Cell Dev Biol. 2021 Nov 15;9:773861. doi: 10.3389/fcell.2021.773861 (PMC8634598; doi:10.3389/fcell.2021.773861)
Supplement: Supplementary file 1 [file DataSheet1.PDF]

SUPPLEMENTARY MATERIAL

I. Code of Exoquant macro with notes

```
//////MEASURING NEURON-EXTERNAL PROTEINS//////

////To select the parameters that are going to be measured////
run("Set Measurements...", "area mean standard modal min center perimeter
integrated median skewness kurtosis area_fraction redirect=None decimal=3");
run("Colors...", "foreground=white background=black selection=yellow");

////To measure the total intensity of each channel////
//It is assumed that green channel is going to be the external protein to be
measured in channel 1//
//It is assumed that red channel (membrane marker) is in channel 2//
Stack.setChannel(2);
hrpgfp = getImageID();
run("Measure");
Stack.setChannel(1);
run("Measure");

////To obtain the perimeter of the neuron from the red channel////
Stack.setChannel(2);
run("Duplicate...", "use");
hrpThreshold=getImageID();
setAutoThreshold("Otsu b&w dark");
run("Convert to Mask");
setTool("Wand");
run("Wand Tool...", "tolerance=0 mode=8-connected");;//Choose over 4-
connected and tolerance 1-5
```

```
waitForUser("Choose the perfect bouton and then click ok");


////To obtain the parameters measured from the inside of the neuron////
selectImage(hrpgfp); //To select again the complete stack
Stack.setChannel(2);
run("Restore Selection");
run("Measure");
Stack.setChannel(1);
run("Restore Selection");
run("Measure");


////To obtain the parameters from outside the neuron to the enlargement size selected////
run("Clear", "stack");//To clean inside the neuron//
Stack.setChannel(2);
run("Enlarge...", "enlarge=1"); //This is 1 micrometer enlargement but if you change the 1 you will obtain diferent size//
run("Measure");
Stack.setChannel(1);
run("Restore Selection");
run("Enlarge...", "enlarge=1");
run("Measure");


////To measure the total intensity of each channel without the neuron inside intensity////
Stack.setChannel(2);
run("Select None");
run("Measure");
Stack.setChannel(1);
run("Select None");
run("Measure");
```

II. Code of Autophagoquant macro with notes

```
//////MEASURING INTERNAL DOT PROTEINS//////


//The results table should be empty because the function getResult will look
into the results table and select the mean of the second line otherwise the
quantification could not be reproducible

run("Clear Results");

run("8-bit");

run("Colors...", "foreground=white background=black selection=yellow");


/////To obtain the size of the surroundings with DLG bouton (1)/////

Stack.setChannel(2);

setAutoThreshold("Otsu dark");

setOption("BlackBackground", false);

run("Convert to Mask", "method=Otsu background=Dark only"); //The otsu method
has been validated from the exosomes experiments

setTool("wand");

run("Wand Tool...", "tolerance=0 mode=8-connected");

waitForUser("Choose the perfect bouton and then click ok");

run("Set Measurements...", "area mean standard modal min center perimeter
integrated median skewness kurtosis area_fraction redirect=None decimal=3");

run("Measure");


Stack.setChannel(1);

run("Duplicate...", "  channels=1");

run("Median...", "radius=1");

run("Measure");

close();
```

```
/////To obtain the mean and the standard deviation from the atg8 channel/////

Stack.setChannel(1);

run("Measure");

//I dont do restore selection because it should be done as it is a stack



/////To obtain the size of atg8 marked buttom/////

run("Clear Outside", "slice");

run("Duplicate...", "duplicate range=1 use");

setAutoThreshold("Otsu dark");

run("Convert to Mask", "method=Otsu background=Dark only");

run("Set Measurements...", "area mean standard modal min center perimeter
integrated median skewness kurtosis area_fraction limit redirect=None
decimal=3"); //The limit command allow just measure inside the selection

run("Restore Selection");

run("Measure");

close();

run("Median...", "radius=1");


/////To obtain the number of autophagosomes/////

//IMPORTANT//

Stack.setChannel(1);

mn = getResult("Mean", 1);

std = getResult("StdDev", 1);

thresholdUnround = mn + (4*std);

threshold = round(thresholdUnround); //it will round the number to the
closest enter number as the threshold option does not allow a number that is
not enter

//if you want to make sure things are going well
//print(mn);//print(std);//print(thresholdUnround);//print(threshold);
```

```
setThreshold(threshold, 255);//(lower, upper threshold level)
run("Convert to Mask", "method=Otsu background=Dark only");
run("Analyze Particles...", "size=0.09-0.50 circularity=0.0-1.00 summarize add slice");

initialParticleNumber = roiManager ("count");
if (initialParticleNumber>1) {
      for (i=0; i<initialParticleNumber; i++){
            roiManager("Select", i);
            run("Enlarge...", "enlarge=0.50");
            roiManager("add");
      };
};

currentParticleNumber = roiManager ("count");
changed = true;

while (changed) {
changed = false;
      for (i = initialParticleNumber; i < currentParticleNumber && !changed; i++) {
            for (j = initialParticleNumber; j < currentParticleNumber && !changed; j++) {
                  if (i < currentParticleNumber) ¨{
                        if (j != i){
                              roiManager ("Select", newArray(j, i));
                              roiManager("and");
                              k = selectionType();
                              //print (k);
                              if (k != (-1)) {
                                    changed = true;
```

```
                                    roiManager ("Select", newArray(j, i));
                                    roiManager("combine");
                                    roiManager("add");
                                    roiManager ("Select", newArray(j, i));
                                    roiManager("delete");
                                    currentParticleNumber = roiManager
("count");
                              };
                        };
                  };
            };
      };
};
```
